# Evolving Paradigm of Radiotherapy for High-Risk Prostate Cancer: Current Consensus and Continuing Controversies

**DOI:** 10.1155/2016/2420786

**Published:** 2016-05-23

**Authors:** Aditya Juloori, Chirag Shah, Kevin Stephans, Andrew Vassil, Rahul Tendulkar

**Affiliations:** ^1^Cleveland Clinic, Taussig Cancer Institute, Department of Radiation Oncology, Cleveland, OH, USA; ^2^Cleveland Clinic, Taussig Cancer Institute, Department of Radiation Oncology, Strongsville, OH, USA

## Abstract

High-risk prostate cancer is an aggressive form of the disease with an increased risk of distant metastasis and subsequent mortality. Multiple randomized trials have established that the combination of radiation therapy and long-term androgen deprivation therapy improves overall survival compared to either treatment alone. Standard of care for men with high-risk prostate cancer in the modern setting is dose-escalated radiotherapy along with 2-3 years of androgen deprivation therapy (ADT). There are research efforts directed towards assessing the efficacy of shorter ADT duration. Current research has been focused on assessing hypofractionated and stereotactic body radiation therapy (SBRT) techniques. Ongoing randomized trials will help assess the utility of pelvic lymph node irradiation. Research is also focused on multimodality therapy with addition of a brachytherapy boost to external beam radiation to help improve outcomes in men with high-risk prostate cancer.

## 1. Introduction

Over 220,000 men are diagnosed with prostate cancer in the United States every year [1]. High-risk prostate cancer is an aggressive form of the disease with a higher risk of distant metastasis and mortality, representing a significant portion of the nearly 28,000 prostate cancer deaths a year [1]. Multiple definitions of high-risk prostate cancer exist with the National Cancer Care Network (NCCN) guidelines defining high-risk prostate cancer as cases with at least one of the following features: Gleason 8–10, clinical stage T3a or higher, or PSA > 20 ng/mL [2]. The use of radiation therapy in the definitive treatment of high-risk prostate cancer has been well studied in multiple prospective randomized trials. It is well understood that local disease control plays an important role in reducing the chance of distant metastasis and cancer-specific mortality [3, 4]. Here we review the current state of external beam radiation therapy (EBRT) for high-risk disease, including the use of androgen deprivation therapy (ADT), the role for hypofractionation and stereotactic body radiation therapy (SBRT), the evolving evidence for combined modality therapy, and controversies regarding pelvic nodal irradiation.

## 2. External Beam Radiation Therapy

An important clinical question in this high-risk population has been whether local therapy provides any benefit in patients that are at an increased risk of distant metastases. This has been addressed by two randomized trials that established the benefit of adding EBRT to androgen deprivation therapy (ADT) alone, outlined in Table 1. Widmark et al. [5] studied 875 patients with intermediate- or high-risk disease randomized to receive ADT + EBRT or ADT alone. The ADT regimen was 3 months of a GnRH agonist followed by continuous antiandrogen (flutamide), and the mean radiation dose was 70 Gy to the prostate and seminal vesicles (SV). The addition of radiation to ADT was shown to improve 10-year overall survival (70% versus 61%, *p* = 0.004), 10-year disease-specific survival (88% versus 76%, *p* < 0.001), and 10-year biochemical-free survival (74% versus 25%, *p* < 0.001), despite a radiation therapy dose that is less than what is currently utilized in the dose escalation era. These results are consistent with a randomized study from Warde et al. [6]. In the Intergroup T94-0110 trial, 1205 patients with high-risk prostate cancer were treated with lifelong ADT, through either bilateral orchiectomy or lifelong luteinizing hormone-releasing hormone (LHRH) agonist. Patients were then randomized to also receive EBRT or not; those treated with radiation received a dose of 65–69 Gy to the prostate and SV. Unlike the Widmark et al. trial, some patients were also treated to the pelvis with mean dose of 45 Gy. The recently published update of the trial [7] demonstrated that the addition of EBRT to ADT significantly improved 10-year overall survival (HR 0.70, 0.57–0.85, *p* < 0.001) and 10-year prostate cancer-specific survival (HR 0.46, 0.34–0.61, *p* < 0.001), again despite lower doses than those used with modern radiotherapy.

These trials provide strong evidence for the use of external beam radiation in these patients; even with lower radiation doses than those currently used, the addition of EBRT provided a 10% survival benefit. Randomized evidence has also demonstrated that conservative treatment with ADT alone provides no benefit compared to observation in this population. Studer et al. [8] examined the use of ADT alone in 985 patients with localized prostate cancer. Patients were randomized to treatment with upfront ADT (bilateral orchiectomy or LHRH agonist) or had ADT reserved until symptomatic disease progression. At median follow-up of 7.8 years, prostate cancer mortality was not significantly improved with upfront use of ADT (19% versus 20%). Thus, local treatment with curative intent is warranted, and the AUA and NCCN recommend the use of definitive radiation in this patient population [2, 9].

## 3. Role for ADT

Multiple randomized trials have demonstrated a benefit in overall survival with the addition of ADT to EBRT in high-risk patients, as shown in Table 2. RTOG 85-31 was among the first trials to establish this benefit [11]. Patients with locally advanced disease (T3 or N1) were treated to the whole pelvis (44–46 Gy) with a 20–25 Gy boost to the prostate. Patients in the RT + ADT arm were treated with an LHRH agonist, goserelin, starting at the end of radiation, while patients in the RT arm were treated with ADT only at the time of disease progression. At 10 years [12], treatment with adjuvant ADT improved overall survival (49% versus 39%, *p* = 0.002) and disease-specific mortality (84% versus 78%, *p* = 0.005). Subset analysis by Gleason score demonstrated that ADT did not provide a survival benefit in Gleason 2–6 patients (57 versus 51%, *p* = 0.26) but did for Gleason 7 (52% versus 42%, *p* = 0.026) and Gleason 8–10 (39% versus 26%, *p* = 0.0046). Disease-specific mortality was only reduced in patients with Gleason ≥8 disease (27% versus 40%, *p* = 0.0039).

RTOG 86-10 was a similar study examining the addition of 4 months of ADT, given prior to and during radiation in patients with bulky disease [13, 14]. Subset analyses demonstrated that, at 8 years, 4 months of ADT improved local and distant control as well as survival in Gleason 2–6 patients. However, in Gleason 7–10 patients, there was no demonstrated statistically significant benefit in any outcome, suggesting that patients with higher risk factors may need longer than 4 months of androgen deprivation to make a notable impact on the natural history of the disease.

EORTC 22863 examined the addition of 3 years of concurrent and adjuvant ADT in patients with prostate-confined disease with high-grade pathology as well as locally advanced patients treated with radiation [17]. At 10 years [18], overall survival (58% versus 40%, *p* = 0.0004) and disease-free survival (48% versus 23%, *p* < 0.0001) significantly improved with addition of ADT.

TROG 96-01 was a three-arm trial including patients with T2b-T4N0 disease treated with radiation and randomized to one of three arms, no ADT, 3 months' ADT, and 6 months' ADT. Randomization was stratified by PSA (greater and less than 20 ng/mL) and grade [16]. Of note, pelvic lymph nodes were not treated in this trial. Local failure, distant failure, and biochemical failure were significantly reduced with use of either 3 or 6 months of ADT compared to patients treated with radiation alone. At ten years, the addition of 6 months of ADT to EBRT alone also reduced distant failure (10.9% versus 20.6%, *p* = 0.0006) and improved overall (70.8% versus 57.5%, *p* = 0.0005) and disease-specific survival (88.6% versus 78%, *p* = 0.0002) [35].

The consensus of the randomized trial evidence suggests that ADT plays a vital role in disease control of high-risk prostate cancer patients. A subset analysis of RTOG 85-31 demonstrated that patients who were treated for longer than 5 years of ADT had the most benefit [36]. Indefinite treatment with androgen deprivation is not without implications on quality of life; thus, it is important to find the optimal length of adjuvant ADT in the curative setting of high-risk patients. One study showed a survival benefit with three years of ADT, while another demonstrated a benefit with only six months of ADT, leaving open the question of duration of treatment needed.

Table 3 summarizes randomized trials that have attempted to help delineate optimal duration by comparing long-term (LTAD) and short-term androgen deprivation (STAD) after radiation. RTOG 92-02 was a large phase III trial in T2c-T4 patients comparing 4 versus 28 months of ADT along with radiation [20]. Use of LTAD significantly improved local and distant disease control, biochemical control, and disease-specific survival at ten years [21]. Only patients in the Gleason 8–10 subset had an overall survival advantage at ten years, 45% versus 32% (*p* = 0.0061). A subsequent cost-analysis of patients included in RTOG 92-02 demonstrated that use of LTAD was associated with increased quality-adjusted life years as well as decreased total costs, due to the salvage treatments associated with STAD [37].

EORTC 22961 showed that 36 months of ADT with EBRT significantly improved overall survival at 5 years (85% versus 81%) compared to 6 months of ADT [19]. As will be discussed, the recent DART 01/05 trial has demonstrated that use of 28 months of ADT is superior to 4 months of ADT in the dose-escalated EBRT era [22]. The body of evidence from these randomized trials shows that patients with high-risk disease have a survival benefit with LTAD. As such, currently the NCCN guidelines for high-risk prostate cancer include 2-3 years of androgen deprivation along with EBRT as a category 1 recommendation [2]. In practice, the optimal duration remains a moving target. In EORTC 22961, for example, 28% of patients in the LTAD arm did not complete the full 3 years of ADT due largely to quality of life factors [19].

A recent Canadian randomized trial [23] including 630 high-risk patients has suggested that ADT duration can potentially be reduced from 36 months to 18 months in this population with no significant difference in overall or disease-specific survival. However, the analysis was not powered for noninferiority; more patients are currently being accrued. The study also required treatment to the pelvic lymph nodes; it is unclear if such reduction in ADT duration would be possible in patients with untreated lymph nodes. The impact on quality of life in cutting the required ADT time by half also remains to be reported. While it has been established that high-risk patients need longer than 6 months of ADT, further work remains to be done in examining the safety and efficacy of reducing ADT duration to less than two years. Off protocol however, the goal should still be for these patients to finish at least a two-year course of androgen deprivation.

Androgen deprivation therapy can be associated with obesity, sexual dysfunction, insulin resistance, bone loss, gynecomastia, fatigue, and lipid abnormalities [38]. Side effects of nonsteroidal antiandrogens can also include diarrhea as well as significant hepatotoxicity. As discussed previously, RTOG 85-31, 86-10, and 92-02 established the survival benefit of addition of ADT to EBRT as well as the need for long-term ADT in high-risk patients (Tables 2 and 3). At ten-year follow-up, analysis of outcomes in the roughly 3000 patients included in the three trials demonstrated that grade 3+ GI and GU late toxicity was not increased with the addition of ADT to radiation [39]. In fact, patients treated with long-term ADT had a significantly reduced rate of grade 3+ GU toxicity compared to patients treated with RT alone.

The role of ADT in potentiating cardiovascular disease has been an active area of study and remains an area of controversy. A pooled analysis of 1,372 patients who participated in 3 prospective randomized trials examining the addition of short-term ADT to radiation demonstrated that, in men 65 and older, use of 6 months of ADT led to shorter time to fatal heart attacks compared to those treated with radiation alone [40]. No such difference was observed in men younger than 65. More recently, Nguyen et al. published a large meta-analysis of 4141 patients from 8 randomized trials of patients with unfavorable-risk prostate cancer treated with and without ADT [41]. The rate of cardiovascular death was not significantly different in patients treated with ADT (11.0% versus 11.2%, *p* = 0.41). In addition, patients treated with LTAD had no increase in rates of cardiovascular mortality compared to patients treated with ADT for 6 months or less. In 4805 patients from 11 trials that reported survival, use of ADT significantly reduced rates of prostate cancer-specific mortality (13.5% versus 22.1%) as well as all-cause mortality (37.7% versus 44.4%) [41]. Patients with high-risk prostate cancer have a significant risk of mortality from prostate cancer and the magnitude of benefit provided by ADT far exceeds the additional risk of CV mortality that may potentially exist, though the Nguyen meta-analysis represents the largest patient group in which this has been studied and showed no increased risk. As such, the American Cancer Society, the American Urological Association, and the American Heart Association recommend use of ADT in these patients without any need for cardiovascular workup or intervention prior to initiation of treatment [42]. Some evidence has suggested that patients with history of MI may be more adversely affected with use of ADT [43]. Prospective research is needed on the cardiovascular implications of ADT use in patients with preexisting coronary artery disease. Patients treated with long-term ADT should be counseled in reducing their cardiovascular risk factors.

## 4. Dose Escalation

Though EBRT has been shown to significantly improve survival outcomes in high-risk patients, the aforementioned randomized trials used doses from 65–70 Gy, not reflective of the modern dose escalation in practice. The advent of intensity modulated radiation therapy (IMRT) has allowed for increasing doses delivered to the prostate while avoiding increased normal tissue toxicity. Multiple trials have demonstrated benefit in biochemical control in patients with low-intermediate-risk prostate cancer treated with doses escalated to 74–79.2 Gy [24, 25, 27, 44, 45]. The largest of these is RTOG 01-26 [45], in which 1,499 patients with Gleason 6 or 7 disease were treated without ADT and randomized to either 70.2 Gy or 79.2 Gy in 1.8 Gy fractions. Patients treated to 79.2 Gy had significantly reduced rates of biochemical failure by the Phoenix definition [46], 26% versus 43% at 7 years.

Similarly, dose escalation in high-risk prostate cancer patients has become commonplace. Zelefsky et al. [47] retrospectively reviewed outcomes in 2,047 patients with clinically localized prostate cancer treated definitively with radiation with doses ranging from 66 to 86.4 Gy. In patients with high-risk features, multivariate analysis demonstrated significant reduction in biochemical failure and distant metastases with higher doses of radiation.  Table 4 outlines the results of three large randomized trials that demonstrate the benefit of dose escalation in high-risk patients [24, 26, 27]. The MD Anderson trial did not include the addition of ADT; patients treated with dose escalation to 78 Gy had a roughly 20 percent benefit in biochemical-free survival at median follow-up of 8.7 years [24]. The Dutch [26] and UK [27] trials included more patients, a higher percentage of whom were categorized as high-risk. All three trials demonstrate that dose escalation improves biochemical control; however, there was no significant improvement in overall survival.

The UK and Dutch trials show that, even in the setting of ADT, there is still significant benefit in biochemical control with dose escalation. As discussed previously and outlined in Table 2, the addition of ADT to radiation has been shown to improve biochemical control and overall survival; however, these studies were done in an era of lower doses (65–70 Gy). The recently published DART 01/05 trial [22] randomized 355 patients with intermediate- or high-risk prostate cancer treated with high-dose radiation (76–82 Gy) to 4 months of neoadjuvant ADT alone or with the addition of 2 years of adjuvant ADT (total duration 28 months). Patients with high-risk disease had a significant benefit in biochemical control, distant disease control, and overall survival. Importantly, there was no noted significant increase in late grade ≥3 GI or GU toxicities. This is the first randomized trial to demonstrate a benefit to long-term ADT in the setting of high-dose radiation and it supports the continued use of ADT along with EBRT in the dose escalation era.

## 5. Impact of Pelvic Radiation

The majority of the discussed randomized EBRT + ADT trials (Tables 2 and 3) included patients treated with pelvic radiation, except for TROG 96-01. The rationale for pelvic irradiation is that a nontrivial proportion of clinically localized high-risk prostate cancer patients have micrometastatic nodal disease that is not otherwise apparent [34]. Elective pelvic radiation increases radiation exposure to the bowel and is associated with increased GI toxicities during and after radiation. Thus, patient selection for pelvic irradiation in this cohort has been somewhat controversial. In the DART trial, the decision of whether or not to include the pelvis in the radiation field was left up to the participating institutions [22].

Table 5 summarizes currently published studies looking at field size. RTOG 94-13 [29–31] included patients with an estimated ≥15% chance of lymph node involvement based on the Roach formula [34]. Patients were randomized to prostate-only or whole pelvic radiation; patients were also randomized to total 4 months of neoadjuvant and concurrent ADT or 4 months of adjuvant ADT. In patients treated with neoadjuvant/concurrent ADT, the use of whole pelvic radiation improved progression-free survival as well as biochemical control. However, in patients treated with adjuvant ADT, outcomes were equivalent irrespective of ADT timing. The authors presented their updated data with 12-year follow-up at ASTRO 2013 and conclude that there may be sequence-dependent biological interactions between the field size and ADT. However, as this was a 2 × 2 designed trial, there has been controversy on how these results should be interpreted. In order to address the remaining questions, RTOG 09-24 is currently accruing patients to further examine the impact of pelvic nodal radiation in a two-arm design. These patients will be treated by current standards, with high-dose radiation (45 Gy to the pelvis followed by boost to the prostate to 79.2 Gy) as well as long-term ADT (32 months).

GETUG-01 was a French randomized trial which did not show a benefit in overall survival or progression-free survival with whole pelvic radiation, though the radiation dose (mean total dose of 68 Gy) is low by modern standards [32] In contrast, Aizer et al. retrospectively demonstrated significant improvement in biochemical control with pelvic RT with use of higher doses (mean 75.6 Gy); however, longer follow-up is needed [33]. A recent National Cancer Data Base analysis [48] of more than 14,000 high-risk patients suggested there was no overall survival advantage with whole pelvic radiation compared to prostate-only EBRT, though there are inherent limitations in a retrospective analysis. Currently there is no consensus recommendation for pelvic radiation in this population, and it should be considered on a case-by-case basis until the results of RTOG 09-24 are available.

## 6. Node-Positive Disease

Patients with clinical or pathologic evidence of nodal disease represent a unique cohort of prostate cancer patients, technically classified as stage IV disease, though unlike those with distant metastases, a potential cure is possible. Thus, some have favored an aggressive multimodality therapy approach. A retrospective study published by Zagars et al. [49] demonstrated that, in patients with pathologically confirmed nodal disease (pN1) after a lymphadenectomy, those treated with prostate EBRT (mean dose of 68 Gy) + ADT had improved freedom from distant metastases and improved overall survival compared to those treated with initial ADT alone when controlling for other disease factors such as Gleason score, initial PSA, and T stage. A portion of patients (18%) included on RTOG 85-31 [11], which demonstrated a benefit to the addition of ADT to RT in high-risk patients, had pathologically node-positive disease. In subset analysis of these pN1 patients, the combination of ADT and RT improved OS and distant disease control compared to those treated with radiation alone [50]. Two large population analyses using SEER have also demonstrated improved overall survival and prostate cancer-specific survival in radiographic and pathologic node-positive patients treated with radiation therapy versus those treated with no local therapy, though these analyses are limited by lack of information regarding ADT [51, 52]. Current guidelines [2] recommend either the combination of long-term ADT and EBRT or long-term ADT alone for node-positive patients, though the evidence suggests a rationale for aggressive combination therapy in these patients. However, there is a dearth of randomized evidence for this population and future studies should focus on the role for ADT with modern radiation doses as well as the role for pelvic nodal radiation in clinically node-positive patients.

There is also controversy regarding the management of pathologically node-positive patients after prostatectomy. Briganti et al. retrospectively compared outcomes in men treated with prostatectomy and lymph node dissection who were found to have positive lymph nodes and were subsequently treated with radiation therapy plus ADT or ADT alone. Ten-year overall survival (86% versus 70%) and prostate cancer-specific survival (74% versus 55%) were significantly improved with the combination of ADT + RT [53]. Recently, Abdollah et al. [54] published a large retrospective analysis of 1107 patients with pN1 disease who were treated with prostatectomy and lymph node dissection and adjuvantly with ADT ± RT. With a median follow-up of 7.1 years, those treated with RT had improved cancer-specific mortality. Further subset analyses identified two patient groups who benefited most from addition of radiation: (1) patients with two positive nodes or less who also had Gleason 7 disease, pT3 disease, or positive margins or (2) patients with 3-4 positive nodes. Conversely, a large population SEER analysis did not show any benefit in overall or cancer-specific survival to the addition of RT to patients with pN1 disease after surgery [55]. In clinical practice, adjuvant radiation is routinely offered to patients with pN1 disease, though randomized evidence is needed with further study warranted specifically in the subgroups identified in the Abdollah analysis.

## 7. Hypofractionation

Though conventionally fractionated EBRT is standard of care by NCCN guidelines in this population, 8 weeks of daily radiotherapy can be logistically challenging for patients, with increased travel costs and opportunity cost with regard to time [56, 57]. Furthermore, radiobiological studies have demonstrated a low alpha/beta ratio for prostate cancer, suggesting that increased fraction size may improve biochemical control without significantly increased toxicity to nearby tissues. Multiple randomized trials have demonstrated excellent biochemical control with acceptable toxicity profiles with hypofractionated courses in low-, intermediate-, and high-risk prostate cancer patients [58–63]. Arcangeli et al. [63] examined 168 patients, all with high-risk disease, randomized to conventional fractionation (80 Gy/40 fractions) or hypofractionation (62 Gy/30 fractions). All patients were treated with 9 months of ADT. No differences in toxicities were noted in the two arms [64]. At 5 years, freedom from biochemical failure (95% hypofractionated versus 83% conventional), local failure (100% versus 92%), and distant failure (98% versus 87%) was statistically equivalent in the two arms. However, in a subset analysis of high-risk patients with PSA < 20 ng/mL, hypofractionation improved all three outcomes.

More recently, the HYPRO trial group randomized 820 patients with intermediate- (27%) and high-risk (73%) prostate cancer to standard (78 Gy in 39 fractions, five fractions a week) or hypofractionated treatment (64.6 Gy in 19 fractions, three fractions a week). Early reporting of oncologic outcomes demonstrates equivalent outcomes in the standard and hypofractionated groups (5-year relapse-free survival 77% versus 80%, *p* = 0.36) [65]. However, 5-year reports of late toxicity data could not demonstrate that hypofractionation was noninferior to standard fractionation, with cumulative grade ≥3 genitourinary toxicity of 19% using hypofractionation (versus 12.9 % in the standard arm) [66]. Grade ≥2 GI acute toxicity was also reported to be worse in the hypofractionated arm (42% versus 31%) though acute GU toxicity was similar in both arms [58]. While the reported toxicity profiles with hypofractionation in this trial were worse than with standard treatment, some have argued that this may be due to lack of quality assurance with use of image guidance as well as lack of bladder dose constraints [67]. Another large scale European hypofractionation trial, the CHHiP study, included a portion of high-risk patients (12%) and randomized 2100 patients to either standard fractionation (74 Gy in 37 fractions) or one of two hypofractionated regimens: 60 Gy in 20 fractions or 57 Gy in 19 fractions [68]. While treatment efficacy has not yet been published, with median follow-up of 50 months, patient-reported outcomes of bowel toxicity are low and not different between standard and hypofractionated treatment groups. Longer follow-up is needed and, in clinical practice, careful patient selection and image guided radiation therapy with strong consideration for use of daily cone beam CT are warranted.

Though pelvic radiation is sometimes warranted in this patient population, only one published randomized trial, a Lithuanian study with 124 patients [69], included patients with hypofractionated regimens to the whole pelvis. 76 Gy in 38 fractions (arm 1) was compared to 63 Gy in 20 fractions (arm 2); the pelvic regimens included were 46 Gy in 23 fractions in arm 1 and 44 Gy in 20 fractions in arm 2. The hypofractionated arm had simultaneous pelvic and prostate treatment. Only acute toxicities have been reported thus far and incidence was found to be roughly equivalent in both arms, though patients undergoing hypofractionated treatment experienced acute toxicity earlier during treatment.

## 8. Role for Brachytherapy

Use of prostate brachytherapy allows for the ability to safely deliver higher biological equivalent dose to the prostate, which provides some theoretical advantages in high-risk prostate cancer patients. Multiple studies have demonstrated the efficacy of high-dose rate (HDR) brachytherapy as monotherapy or in conjunction with external beam radiation [70–73]. In a phase II trial of 200 high-risk and very high-risk patients, patients were treated with 54 Gy to the prostate and pelvic lymph nodes followed by 19 Gy to the prostate in four HDR treatments. Five-year results demonstrated 85.1% biochemical relapse-free survival without significant increase in toxicity. There is also randomized evidence suggesting a benefit to multimodality therapy with use of low dose rate (LDR) brachytherapy. The results of a prospective randomized trial were recently presented by Morris et al. at ASCO in 2015 [74]. In this trial, 400 patients with intermediate- and high-risk disease were given an LHRH agonist for 8 months and then treated to the whole pelvis with 46 Gy in 23 fractions via EBRT; patients were then randomized to receive 32 Gy/16 fractions conformal EBRT boost or LDR-brachytherapy boost prescribed to minimum peripheral dose of 115%. The 9-year biochemical failure-free survival was 83% with use of LDR boost compared to 63% with external beam boost (HR 0.35, 95% 0.19–0.65; *p* < 0.001). These excellent results strongly support the consideration for dose escalation with multimodality therapy in high-risk patients. Patients with high volume disease and high Gleason score should be considered for this option of combined modality therapy.

## 9. Stereotactic Body Radiation Therapy (SBRT)

Stereotactic body radiation therapy (SBRT) to the prostate represents an ultrahypofractionated regimen, providing definitive treatment, typically in 4–6 fractions. The initial phase 1 dose escalation studies were performed predominantly in low- and intermediate-risk patients [75], but prospective phase II studies have since been done that also included a small proportion of high-risk patients. A pooled multi-institutional analysis of 1100 patients (58% low-risk, 30% intermediate-risk, and 11% high-risk) treated with a median dose of 36.25 Gy in 4-5 fractions demonstrated 5-year biochemical recurrence-free survival of 95%, 84%, and 81% in low-, intermediate-, and high-risk patients [76]. Long-term quality of life measures in patients evaluated for 5 years showed an initial decline in urinary and bowel function within the first three months; however, these were found to return to baseline by six months [77]. Sexual decline was typically noted in the first nine months and then stabilized before declining by typical age-expected parameters.

There is limited data on the use of SBRT in high-risk patients alone. Given the inferior biochemical control after SBRT reported in patients with high-risk disease compared to those with low- and intermediate-risk disease, there have been attempts to dose-escalate. A recently published phase I/II trial examined the use of SBRT in high-risk patients with dose escalation to 40 Gy in 5 fractions along with 1 year of ADT [78]. Uniquely, this trial included treatment to pelvic nodes as well (25 Gy to the pelvic nodes and 40 Gy to the prostate in five total fractions). Four of the 15 patients treated had grade 3 or higher GI or GU toxicity at six months, and the trial was closed early. In the coming years there will be multiple published reports of experiences with use of SBRT in high-risk patients. As this modality becomes more established, it will be imperative to determine the appropriate use of ADT and role of pelvic lymph node irradiation with SBRT.

Boike et al. [75] also reported increased toxicities in their prospective dose escalation study for low- and intermediate-risk patients who were treated in cohorts of 45 Gy, 47.5 Gy, and 50 Gy in 5 fractions. 7% of patients experienced grade ≥3 GI toxicity with 5 requiring a diverting colostomy (250). Based on these two studies, there has been concern about the safety of uniform prostate dose escalation and some have explored more heterogeneous techniques. Kotecha et al. recently reported outcomes in patients with intermediate- and high-risk prostate cancer treated with dose escalation utilizing a novel heterogeneous planning technique. Dosing was 36.25 Gy in 5 fractions with simultaneous integrated boost to 50 Gy in 5 fractions. 3 mm expansions around the urethra, rectum, and bladder were limited to 36.25 Gy with the rest of the gland treated to a mean dose of 50 Gy. With a median two years of follow-up, the 24 treated patients (13 high-risk) had 96% biochemical control (using the Phoenix definition) with no acute or late grade ≥3 GI or GU toxicities noted. Sixteen patients (67%) were treated with ADT for a median of six months. Testosterone levels were monitored regularly and at last follow-up, all patients were no longer castrate except for two undergoing long-term ADT (>24 months). Though longer follow-up is needed, the demonstrated excellent biochemical control in the setting of noncastrate levels of testosterone suggests that this heterogeneous dose escalation technique may represent a safe and efficacious model for treatment.

Another approach under study is SBRT utilizing dose escalation to visible prostate lesions seen on MRI, as opposed to previously published reports using homogenous dose escalation or the urethral sparing heterogeneous dose escalation technique published by Kotecha et al. [79]. This idea has been explored using conventional IMRT, with early reports demonstrating safety with boosting dose to visible MRI lesions to 80 Gy [80] or 95 Gy [81], though efficacy using this technique has yet to be demonstrated. Another recently reported approach utilized HDR brachytherapy boost to MRI lesions after hypofractionated external beam radiation therapy with good tolerance and excellent early toxicity profiles [82]. However, there is some concern regarding the efficacy of these techniques because it is unknown what the relationship is between a dominant lesion on imaging and the true biology of the disease. Some have argued that, because of the potentially multifocal nature of prostate cancer, it is important to maintain adequate whole organ dose in the setting of partial dose escalation. For example, some have performed partial brachytherapy to target the peripheral zone as delineated by MRI with the rationale that this area represents the most common site of prostate cancer [83]. However, this approach was shown to have inferior outcomes in men with favorable intermediate-risk cancer compared to traditional techniques. SBRT with a focal boost to MRI-visible lesions has been reported in low- and intermediate-risk patients; Aluwini et al. reported on 50 patients treated to 38 Gy in 4 fractions with a simultaneous boost to 44 Gy in 4 fractions for the MRI lesion. Biochemical control was excellent (100%) at two years with acceptably low toxicity [84]. Institutional studies using a similar focal dose escalation technique to MRI lesions in high-risk patients are currently accruing.

## 10. Conclusions

The combination of long-term ADT and external beam radiation in high-risk prostate cancer patients has been shown in multiple randomized trials to maximize disease control and extend overall survival compared to single modality treatment. Current recommendations are for 2-3 years of ADT and dose-escalated RT to the prostate. Newly presented randomized data suggests that dose escalation with use of LDR-brachytherapy boost may be superior to dose escalation with EBRT alone. As we enter a new era of healthcare economics, it will be increasingly important to provide appropriate care while using fewer resources, and hypofractionation will almost certainly play a role. While results of long-term follow-up are needed, randomized trials have shown good efficacy with acceptable toxicity with significant reduction in treatment times. In the coming years, more randomized data utilizing hypofractionated regiments as well as SBRT will be available to help shape the guidelines. The decision of whether to target pelvic lymph nodes with radiation remains an unanswered question; results from RTOG 09-24 will help radiation oncologists counsel patients in regard to weighing the increased toxicities against the potential benefits.

## Figures and Tables

**Table 1:** Randomized trials examining the addition of radiation to ADT for high-risk patients.

Trial	Study cohort	Median follow-up	Trial arms	Outcomes	Toxicity
Intergroup T94-0110 Warde et al. [6, 7, 10]	1205 patients (1057 with T3-T4 disease)	8 years	ADT versus ADT + RT (65–69 Gy) ADT: lifelong LHRH agonist or bilateral orchiectomy	10-year OS (45% versus 55%, *p* = 0.001)	EBRT increased bowel, urinary, and sexual dysfunction at six months, but no difference at 3 years

SPCG-7 Widmark et al. [5]	875 patients T1b-T2 G2-G3 or T3 (78%) and PSA < 70, N0	7.6 years	ADT versus ADT + RT (median 70 Gy) ADT: 3 months' GnRH agonist followed by continuous antiandrogen	10-year OS (61% versus 70%, *p* = 0.004) 10-year DSS (76% versus 88%, *p* < 0.001)	RT arm with slightly increased rates of late urinary, GI, and sexual dysfunction at 4 years. Quality of life scores equal at 4 years

**Table 2:** Randomized trials examining the addition of ADT to radiation for high-risk patients.

Trial	Study cohort	Median follow-up	Trial arms	Outcomes
RTOG 85-31 [11, 12]	945 patients T3 (82%) or N1 (18%)	7.6 years	RT versus RT + ADT (44–46 Gy to whole pelvis; 20–25 Gy boost to prostate) ADT: goserelin at least 2 years, preferably until progression	10-year OS (39% versus 49%, *p* = 0.002) 10-year DSS (78% versus 84%, *p* = 0.005) Overall survival benefit limited to patients with Gleason 7–10

RTOG 86-10 [13–15]	456 patients T2-T4, N0-1 with “bulky” disease (palpable ≥ 25 cm^2^)	11.9 years	RT versus RT + ADT (44–46 Gy to whole pelvis; 20–25 Gy boost to prostate) ADT: 4 months' goserelin + flutamide, starting 2 months prior to RT	10-year OS (34% versus 43%, *p* = 0.12) 10-year DSS (23% versus 36%, *p* = 0.01) Subset analyses at 8 years showed that benefit was confined to Gleason 2–6 patients. No benefit to ADT in Gleason 7–10

TROG 96-01 [16]	802 patients T2b-T4N0	10.6 years	RT alone versus RT + 3 mo. ADT versus RT + 6 mo. (66 Gy, no pelvic node treatment) ADT: goserelin + flutamide given *neoadjuvantly*	At 10 years, addition of 6 months' ADT improved 10-year OS (70.8% versus 57.5%, *p* = 0.0005) 10-year DSS (48% versus 23%, *p* < 0.0001)

EORTC 22863 [17, 18]	415 patients T1-2N0 grade 3 or T3-4N0-1	9.1 years	RT versus RT + 3 years' ADT (50 Gy to pelvis, 20 Gy boost) ADT: 1 month' cyproterone acetate, goserelin × 3 years starting with RT	10-year OS (40% versus 58%, *p* = 0.0004) 10-year DSS (10% versus 30%, *p* < 0.0001)

OS: overall survival, DSS: disease-specific survival.

**Table 3:** Randomized trials comparing LTAD and STAD with radiation in high-risk patients.

Trial	Study cohort	Median follow-up	Trial arms	Outcomes
EORTC 22961 [19]	970 patients with T2c-T4 or N1-2	6.4 years	RT + 6 months' ADT versus RT + 36 months' ADT (Prostate dose 70 Gy) ADT: 6 months' CAB (LHRH agonist + antiandrogen) ± 2.5 years' LHRH agonist	5-year OS 81% versus 85% (*p* = 0.02) 5-year DSS 95% versus 97% (*p* = 0.002) QOL measures the same in each arm No difference in cardiac fatal event Increased rates of reported gynecomastia, incontinence, and sexual dysfunction with LTAD

RTOG 92-02 [20, 21]	1514 patients with T2c-T4	11.3 years	RT + 4 months' ADT versus RT + 28 months' ADT (44–50 Gy to whole pelvis, boost to 65–70 Gy prostate) ADT: goserelin + flutamide 4 months total (prior to and during RT) ± 2 years' goserelin	10-year OS 52% versus 54% (*p* = 0.25) 10-year DSS 84% versus 89% (*p* = 0.0001) Gleason 8–10 subset: 10-year OS 32% versus 45% (*p* = 0.0061) Increased grade 3 GI toxicity at 8 years with LTAD (2.9% versus 1.2%, *p* = 0.04)

DART 01/05 Spain [22]	355 patients (47% int.-risk, 53% high-risk)	5.3 years	RT + 4 months' ADT versus RT + 28 months' ADT (76–82 Gy to prostate) ADT: goserelin + antiandrogen for 4 months total (prior to and during RT) ± 2 years' goserelin	5-year OS 86% versus 95% (*p* = 0.009) 5-year BRFS 81% versus 89% (*p* = 0.019) 5-year metastasis-free survival 83% versus 94% (*p* = 0.009)

PCS IV Trial Canada Nabid et al. [23]	630 node-negative, high-risk patients	6.5 years	RT + 18 months' ADT versus RT + 36 months' ADT (44 Gy to whole pelvis, 70 Gy to prostate) ADT: bicalutamide 1 month, goserelin q 3 months for 18 or 36 months	10-year OS 59% versus 62% (*p* = 0.28) 10-year DSS 84.1% versus 83.7% (*p* = 0.82)

LTAD: long-term ADT, STAD: short-term ADT, OS: overall survival, DSS: disease-specific survival, and BRFS: biochemical relapse-free survival.

**Table 4:** Randomized trials studying dose escalation in high-risk patients.

Trial	Study cohort	Median follow-up	Trial arms	Outcomes
MDACC Kuban et al. [24]	301 patients 20% low-risk 46% int.-risk 34% high-risk	8.7 years	70 Gy versus 78 Gy 4-field box or 3DRT techniques No ADT used	8-year BRFS 55% versus 78% (*p* = 0.004) 8-year OS 78% versus 79% (NS) High-risk cohort: 8-year BRFS 26% versus 63% (*p* = 0.004) 1% versus 7% grade 3 late toxicity (*p* = 0.02)

Dutch [25, 26]	664 patients T1b-4 18% low-risk 27% int.-risk 55% high-risk	5.8 years	68 Gy versus 78 Gy 3DRT technique ADT used	7-year BRFS 45% versus 56% (*p* = 0.04) OS not significantly different Late grade 3+ GI (4% versus 5%) and GU toxicity (12% versus 13%) equivalent in both arms

UK MRCRT01 [27, 28]	843 patients 19% low-risk 37% int.-risk 43% high-risk	10 years	64 Gy versus 74 Gy 3DRT technique ADT used	10-year BRFS 43% versus 55% (*p* = 0.0003) OS not significantly different 6% versus 10% grade 3 late toxicity

BRFS: biochemical relapse-free survival, NS: nonsignificant, and OS: overall survival.

**Table 5:** Pelvic nodal radiation in high-risk patients.

Study	Study cohort	Median follow-up	Trial arms	Outcomes
RTOG 94-13 [29–31]	1275 patients, 73% Gleason 7–10	12 years	PRT NA/C ADT + pelvic RT NA/C ADT + prostate RT Adjuvant ADT + pelvic RT Adjuvant ADT + prostate RT	Significant improvement in biochemical control, trend for improved progression-free survival with use of NA/C ADT + pelvic RT

GETUG-01 [32]	444 patients, T1b-T3N0 (75% high-risk)	3.5 years	PRT Prostate RT versus pelvic RT prostate boost 46 Gy to the pelvis, 66–70 Gy to the prostate	No difference in PFS or OS with use of pelvic node radiation No significant difference in toxicity or QOL measures

Yale Aizer et al. [33]	277 patients with ≥15% LN involvement per Roach formula [34]	2.5 years	Retrospective review: Whole pelvic RT/prostate boost versus prostate RT alone ≥90% received ADT Mean RT dose: 75.6 Gy	4-year biochemical-free survival improved with pelvic RT (86% versus 70%, *p* = 0.02) in multivariate analysis OS not reported Increased acute GI toxicity with pelvic RT, no difference in late toxicity

PFS: progression-free survival, OS: overall survival, PRT: prospective randomized trial, and NA/C: neoadjuvant/concurrent.
